# Nanosponge-integrated hydrogel of *Vranaviropana taila*: bridging Ayurveda and nanotechnology for enhanced wound healing in primary care

**DOI:** 10.3389/fphar.2026.1747380

**Published:** 2026-04-10

**Authors:** Remya Rajan, Dhanya Soman Pillai, Arun Mohanan

**Affiliations:** Department of Rasashastra and Bhaishajya Kalpana (Pharmaceuticals), Amrita School of Ayurveda, Amritapuri, Amrita Vishwa Vidyapeetham, Kollam, Kerala, India

**Keywords:** antimicrobial, ayurveda, hydrogel, L929 fibroblast, lepa kalpana, nanosponge, *Vranaviropana taila*, wound healing

## Abstract

**Introduction:**

Effective wound management remains a significant challenge in primary healthcare, especially in resource-limited settings where accessible and cost-effective dressings are predominant. *Vranaviropana taila*, a traditional Ayurvedic formulation, offer therapeutic potential; however, their clinical utility is often inhibited by suboptimal drug delivery. This study aimed to develop and evaluate a novel nanosponge-integrated hydrogel drug delivery system for *Vranaviropana taila* to strengthen its wound healing efficacy for primary healthcare applications.

**Methods:**

The phytopharmacological analytical tests of *Vranaviropana taila* reported the presence of flavonoid and phenol rich compounds with strong antioxidant potential, supporting the traditional use of formulation in tissue repair. Using emulsion solvent diffusion method nanosponges containing the oil were prepared and subsequently incorporated into a carbopol hydrogel matrix. The nanosponges were characterized for zeta potential, polydispersity index (PDI) and particle size. The final hydrogel formulation was assessed for *in vitro* wound healing efficacy using L929 fibroblast models (scratch assay and collagen deposition) along with its biocompatibility (MTT assay), swelling behavior and its *in vitro* drug release capacity by diffusion studies.

**Results:**

The oil-loaded nanosponges exhibited a zeta potential of −6.96 mV and a narrow PDI of 0.061 with uniform average particle size of 461.3 nm. The nanosponge-integrated hydrogel possessed favorable dressing properties, including sustained *in vitro* release and a swelling index of 57.5% and it was biocompatible with a cell viability of 91.96%. In the 72-h of the scratch assay, the nanosponge-hydrogel group demonstrated significantly accelerated wound closure of 96.5%, compared to both the *Vranaviropana taila* group with 89.4% closure and the untreated control (hydrogel). Furthermore, superior to the 21.41% increase observed with the plain oil, the hydrogel formulation induced a 44.19% increase in collagen deposition.

**Conclusion:**

This study successfully integrates a traditional Ayurvedic remedy with a modern nanobiotechnology platform. Compared to the traditional oil *Vranaviropana taila* nanosponge-hydrogel matrix demonstrates enhanced *in vitro* wound healing efficacy, superior collagen production, and optimal physico chemical properties. This formulation represents a promising, cost-effective, and accessible wound care solution, holding significant potential for translation into primary and community healthcare settings.

## 1 Introduction

In primary healthcare, wound management represents a significant clinical and economic challenge, particularly in resource-limited settings where advanced dressings are often inaccessible or prohibitively expensive. Polyherbal formulations, specifically medicated oils (*taila*), are reported to have phytochemical constituents with potent wound healing, anti-inflammatory, and antimicrobial properties. Therefore, traditional medical systems, such as Ayurveda, utilize numerous such formulations for tissue repair. They have long been practised for the management of wounds, ulcers, and inflammatory skin disorders. Though they have reported therapeutic relevance, conventional oil-based formulations have significant limitations, including reduced patient compliance due to their messy application, poor dermal adherence, and high lipophilicity. These physical drawbacks collectively restrict the translational applicability of these valuable formulations in modern wound care settings.

The emergence of nanotechnology (Rarokar et al., 2024; Sangare et al., 2024; Tehri et al., 2022) - enabled drug delivery platforms offer an effective strategy to enhance the clinical utility of lipophilic bioactives. Due to their high entrapment efficiency, large surface area, and ability to sustain drug release, Nanosponges (Tiwari and Bhattacharya, 2022; Aneja et al., 2025; Pyrak et al., 2025; Selvamuthukumar et al., 2012; Khan et al., 2016; Bhowmik et al., 2018)—porous, crosslinked polymeric systems—improve the solubility, stability, and skin permeation of encapsulated bioactives. When dispersed in hydrogel matrices (Ho et al., 2022; Mohanram et al., 2021), these carriers offer additional advantages, including ease of topical application, cooling effects, and hydration, which make nanosponge-integrated hydrogels (Sahiner et al., 2016; Kamoun et al., 2017; Rezakhani et al., 2024; Lin and Hsu, 2025; Di Franco et al., 2025) a promising class of wound dressings.

In this study, a nanosponge-based hydrogel system incorporating *Vranaviropana taila*, (Baldim et al., 2022), a phytoconstituent-rich oil derived from *Euphorbia neriifolia* (Chang et al., 2023a) and *Calotropis gigantea* (Sivapalan et al., 2023), was developed and systematically evaluated. The latex of *E. neriifolia* (*Snuhi*) contains triterpenoids and flavonoids with anti-inflammatory activity (Chang et al., 2023b), while *C. gigantea* (*Arka*) latex exhibited significant wound healing and antimicrobial properties (Ahmed et al., 2021). Sesame oil, acting as a lipid vehicle, combines with these phytoconstituents to form a bioactive system which supports tissue repair, fibroblast proliferation, and collagen deposition (Sivapalan et al., 2023; Ahmed et al., 2021; Deol et al., 2024).

The novelty of this work derives from both the engineering of the delivery system and the specific phytopharmacological selection. Conventional synthetic wound management agents—such as silver sulfadiazine or synthetic antibiotics—are often associated with delayed re-epithelialization, global threat of antimicrobial resistance and cytotoxicity. Unlike this, *Vranaviropana taila* and its hydrogel form, offers a synergistic, multi-targeted therapeutic approach. Without the adverse effects typical of chemical agents, the combination of *Snuhi* and *Arka* provides simultaneous anti-inflammatory, antimicrobial and antioxidant efficacy. Furthermore, this study addresses a unique formulation challenge distinct from simple herbal extracts: the encapsulation of a complex, lipophilic *taila*. Soft lipid-based carriers such as solid lipid nanoparticles (SLNs), liposomes or nanoemulsions often demonstrate poor low entrapment efficiency for heavy, multi-component oils, drug leakage and thermodynamic stability (Ghasemiyeh and Mohammadi-Samani, 2018). Dissimilar to them, the cross-linked, highly porous architecture of ethyl cellulose nanosponges ensures higher loading capacity and superior structural integrity. Furthermore, while existing nanosponge-hydrogel literature predominantly focuses on the delivery of single-molecule synthetic drugs, our approach establishes the sustained release and stabilization of a crude, polyherbal traditional lipid formulation (Zhang et al., 2022). This study also employs a specific excipient strategy to transform a low-compliance, greasy, traditional remedy into a non-staining, hydrophilic, and sustained-release dressing, by utilizing ethyl cellulose—a biocompatible polymer (Allahyari et al., 2026) to fabricate nanosponges and integrating them into a Carbopol hydrogel matrix. This engineered compatibility represents a novel bridge platform, offering a safer and more accessible alternative to synthetic dressings.

A clear research gap exists in bridging the therapeutic potential of this traditional oil with modern pharmaceutics. Even though the properties of *Vranaviropana taila* are established in classical texts and preliminary studies, no investigation has yet addressed its significant formulation-based limitations by integrating it into an advanced nanocarrier system.

Therefore, this study aimed to develop, formulate, and systematically evaluate a novel *Vranaviropana taila*-loaded nanosponge-integrated hydrogel. This integrative approach aims to establish a biomacromolecular platform that combines traditional phytopharmacology with advanced nanocarrier technology for enhanced wound repair and infection control in primary health care.

## 2 Materials and methods

### 2.1 Pharmaceutical study

#### 2.1.1 Pharmaceutical preparation of *Vranaviropana taila*

Vranaviropana taila was prepared according to the Ayurvedic classical textbook *Sarangadhara Samhita* (Shastri, 2013), following the traditional methodology to facilitate the extraction of bioactive constituents into the lipid base. The ingredients comprised latex extracts of *E. neriifolia* (*Snuhi*) and *C. gigantea* (*Arka*) as phytoconstituent sources; sesame oil (*tila taila*) as the lipid vehicle; water as the aqueous phase; and beeswax (*Madhuchishta*) as a stabilizer (Table 1).

**TABLE 1:** Composition of *Vranaviropana taila*.

Ingredient	Proportion (part)
*Snuhi* latex (*Euphorbia neriifolia*) + *Arka* latex (*Calotropis gigantea*)	1/4
Sesame oil (*Tila taila*)	1
Water	4
Beeswax (*Madhuchishta*)	1/8

**Method of preparation** Sesame oil was gently heated with the latex extracts and water in a stainless-steel vessel under continuous stirring till traditional indicators of completion (*siddha lakṣaṇas*), such as wick-like consistency of the herbal residue, uniform frothing at the oil surface and disappearance of the aqueous phase, were observed. This phase corresponds to the endpoint of aqueous evaporation and the transfer of phytoconstituents into the lipid phase. The hot oil was immediately filtered into a vessel containing pre-weighed beeswax and stirred until complete dissolution. The mixture was filtered again and, after cooling, stored in airtight amber glass containers to minimize photodegradation.

#### 2.1.2 Preparation of taila-loaded nanosponges

Taila-loaded nanosponges were prepared using the emulsion–solvent evaporation technique (Zhang et al., 2025), a method widely used for ethyl cellulose-based nanosystems. For the organic phase, 2 mL of *Vranaviropana taila* were dissolved in 10 mL of dichloromethane containing 10% w/v ethyl cellulose (EC). The aqueous phase, consisting of 15 mL of a 10% w/v solution of polyvinyl alcohol (PVA), was mixed dropwise with the organic phase (specifically, the prepared disperse phase was dropped slowly into the aqueous solution, maintaining an EC:PVA ratio of 1:1). After 30 min of constant stirring at 1,200 rpm, nanosponge dispersions were formed. Following controlled evaporation, the suspension was poured into a Petri dish and dried for 24 h at 40 °C in an oven. The dried nanosponges were collected and stored in a desiccator to remove residual solvent and moisture.

#### 2.1.3 Formulation of nanosponge-integrated hydrogel

**FIGURE 1:**
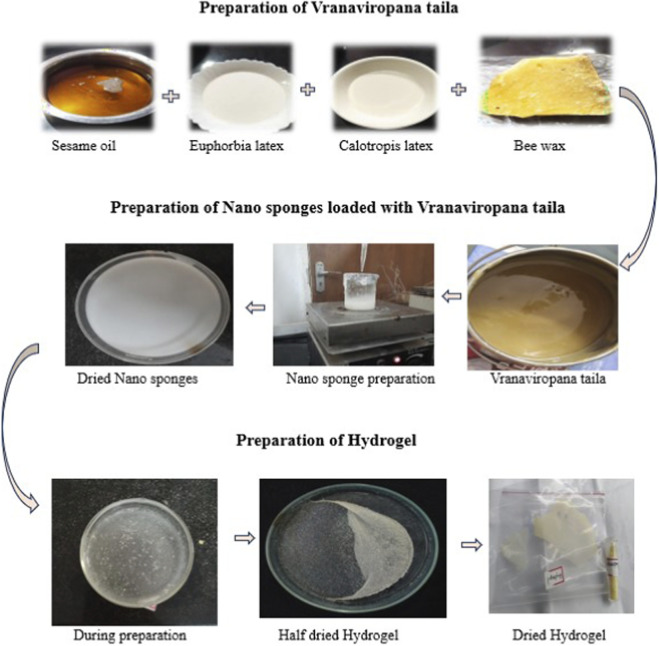
Schematic illustration of Vranaviro pana taila preparation, followed by fabrication of taila-loaded nanosponges and their incorporation into a hydrogel matrix to obtain the final topical formulation.

The dried nanosponges (0.5 g of taila-loaded nanosponges) were mixed into a Carbopol 940 hydrogel matrix (Ostovar et al., 2023), to improve topical application and wound site retention. Carbopol 940 (0.4% w/v) was dissolved in 5 mL of double-distilled water and left to hydrate (swell) overnight. The nanosponge dispersion was gradually added to the hydrated polymer (specifically, the swollen Carbopol was added to the 0.5 g nanosponge dispersion) and stirred continuously at a speed of 1,200 rpm for an additional 10 min (within a 40-min total timeframe) to achieve uniform distribution and allow the formation of the Carbopol hydrogel integrating the taila nanosponges. The formed hydrogel was allowed to stand for 15 min without stirring. After this incubation, the hydrogel was poured into a Petri dish and allowed to dry. The final preparation was transferred to sterile, screw-capped glass containers and stored at 4 °C until further use (Figure 1).

### 2.2 Characterization of *Vranaviropana taila*

Following the Ayurvedic Pharmacopoeia of India (API) guidelines, the prepared oil was evaluated for organoleptic characteristics such as colour, odor, and state, physicochemical parameters such as loss on drying (Niksic et al., 2021), specific gravity (The Ayurvedic Pharmacopoeia of India, 2010, p:193), refractive index at 25 °C (21, p:193), acid value (21, p:201), iodine value (21, p:200), peroxide value (21, p:201), TLC and HPTLC.

#### 2.2.1 TLC (thin layer chromatography)

**FIGURE 2:**
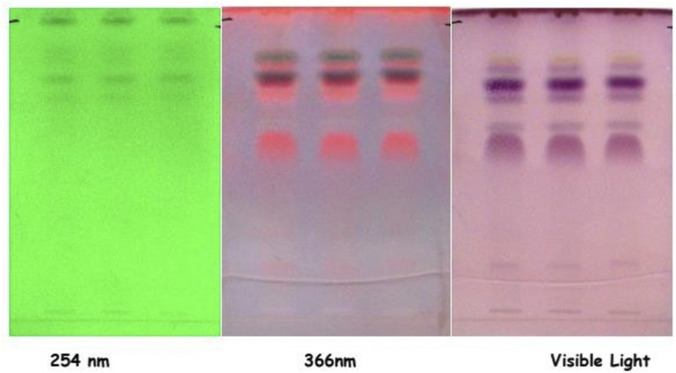
Thin-layer chromatography (TLC) fingerprint profile of Vranaviro pana taila developed on silica gel 60 F254 plates using an optimized mobile phase, visualized under UV light at 254 nm and 366 nm and documented under visible light after derivatization to characterize its phytochemical constituents.

A 1.1134 g *taila* sample was extracted in 5 mL methanol, vortexed, centrifuged (3000 rpm, 5 min), and the supernatant refrigerated. For TLC analysis, a 5 μL aliquot was developed 8 cm using toluene:ethyl acetate:hexane (6:3:1, v/v/v). Post-drying, chromatograms were visualized under UV light (254/365 nm) and derivatized with anisaldehyde-sulphuric acid (105 °C, 10 min) to determine retention factor (Rf) values (Figure 2).

#### 2.2.2 HPTLC (high performance thin layer chromatography)

**FIGURE 3:**
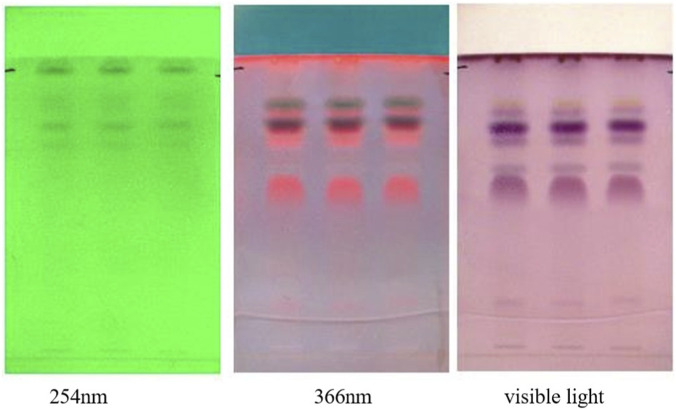
HPTLC fingerprint profile of Vranaviro pana taila developed on silica gel 60 F254 plates using an optimized solvent system, with densitometric documentation under UV light at 254 and 366 nm and visualization under visible light after derivatization to characterize the phytochemical constituents.

HPTLC (High Performance Thin Layer Chromatography) (Sonewane et al., 2025) was performed by spotting the prepared sample (1 mL of *taila* with 2 mL Hexane) on pre-coated silica gel aluminium plate at 15mm, 30mm and 45 mm. After development, Densitometry scanning was performed with a CAMAG Visualizer with a digital camera type DXA 252 at 366 nm to detect phytoconstituents such as flavonoids, terpenoids, and steroids (Figure 3).

### 2.3 Characterization of taila-loaded nanosponges

The surface morphology of the dried, nanosponges was examined using a scanning electron microscope (SEM) under high-vacuum conditions (Ullah et al., 2025; Tehri et al., 2022). Mean particle size and polydispersity index (PDI) were calculated using dynamic light scattering (DLS). Zeta potential was analyzed to evaluate colloidal stability (Muneer et al., 2023). XRD analysis (Hani et al., 2024; Abu-Dief et al., 2020) was conducted to compare plain ethyl cellulose, unloaded nanosponges, and *taila*-loaded nanosponges. (Abdel-Rahman et al., 2022). The disappearance or reduction of crystalline peaks was interpreted as evidence of successful encapsulation and amorphization. Following the extraction of the entrapped fraction through controlled matrix dissolution in dichloromethane, the encapsulation efficiency (EE%) and drug loading (DL%) were calculated by determining the mass of sequestered *Vranaviropana taila* relative to the initial lipid input and the total recovered nanosponge weight.

#### 2.3.1 Scanning electron microscope (SEM)

The microscopic characteristics (shape and morphology) of prepared nanosponges were determined by SEM examination (Ullah et al., 2025). Images were acquired using scanning electron microscopy in various magnifications after nanosponges were made and dried properly to remove moisture content. The powder sample was mounted on metallic stub by using double sided carbon tape, sputter coated with gold by Hitachi E 1010 ion sputter (Japan) and finally imaged using FEI Quanta 200 (Netherland) SEM.

#### 2.3.2 Dynamic light scattering (DLS)

The average mean diameter and size distribution of loaded nanosponges is found by Dynamic Light Scattering method using Malvern zeta sizer at 25 °C. Dried nanosponges were dispersed in water to obtain proper light scattering intensity at 25 °C and sample was placed in disposable sizing cuvette at a count rate of 372.0 (Kcps) for 20s.

#### 2.3.3 Zeta potential

Surface charge is measured by the zeta potential. Using a Zeta sizer (Malvern Instrument) with zeta cells, a polycarbonate cell with gold plated electrodes, and water as the sample preparation medium, the surface charge (electrophoretic mobility) of nanosponge can be evaluated. It is crucial for determining the nanosponge stability.

#### 2.3.4 Xray diffraction analysis

X-ray diffraction analysis of the selected formulation was carried out in an x-ray diffractometer (D8 Advance, Bruker) with Cu Kα radiation (λ = 1.54060 A°). The rate of scanning was 100/min and diffraction angle 2Ɵ was 10–800.

### 2.4 Characterization of nanosponge-loaded hydrogel

#### 2.4.1 Equillibrium swelling study

The swelling capacity of the hydrogel was assessed in phosphate-buffered saline (PBS, pH 7.4) at predetermined time intervals up to 6 h. The swelling index was expressed as the percentage of water uptake (Alagusundaram et al., 2023). *In vitro* drug release studies were conducted using a dialysis membrane diffusion method in PBS at pH 6.8 and 7.4, mimicking skin and wound exudate conditions, respectively. Samples were collected at predetermined intervals, and their absorbance was measured spectrophotometrically at 266 nm for study at pH 6.8 and at 272 nm for the study carried out in phosphate buffer pH 7.4 to quantify the release of phytoconstituents.

#### 2.4.2 Invitro drug release

The *in vitro* release study of nanosponge loaded hydrogel was carried out using spectrophotometric method. 100 mg of hydrogel was dipped in 9 mL of phosphate buffer pH 6.8 (pH of normal skin) and phosphate buffer pH 7.4 (physiological pH) as dissolution medium separately, the test tubes were kept in shaker. 1mL of sample was withdrawn at predetermined intervals and replaced by its equivalent volume of fresh dissolution medium to maintaining the sink condition. The withdrawn aliquots were filtered and read at 266 nm for study at pH 6.8 and at 272 nm for the study carried out in phosphate buffer pH 7.4. The release was represented in terms of OD units.

#### 2.4.3 Spreadability

Spreadability of the nanosponge-integrated *Vranaviropana taila* hydrogel was determined using the glass slide method. A fixed quantity of gel was placed between two glass slides and subjected to a 20 g weight. The test was performed in triplicate at room temperature and the time required for the upper slide to move 5 cm was recorded.

#### 2.4.4 Gelling capacity

Gelling capacity was evaluated by dispersing the nanosponge-loaded hydrogel in phosphate-buffered saline of pH 7.4. The temperature was maintained at 37 °C ± 0.5 °C to simulate physiological conditions. Gel formation time was visually recorded, and gel strength was assessed by gentle agitation to evaluate resistance to deformation.

### 2.5 *In Vitro* studies

#### 2.5.1 Cytotoxicity evaluation

##### 2.5.1.1 Cytotoxic effect of oil by MTT assay

To assess the cytocompatibility of *Vranaviropana taila* by MTT Assay for Oil Sample (Ghasemi et al., 2021; Othman et al., 2025; Niksic et al., 2021; Semenova et al., 2022; Al-Naqeb et al., 2025; Soltanian et al., 2019) L929 fibroblasts (21, p:193), cells were seeded in 96-well plates and treated with serial dilutions of *taila* (12.5–100 μg/mL) for 24 h. After treatment, MTT reagent was added, and the plates were incubated for 3 h. Formazan crystals were dissolved using DMSO, and absorbance was measured at 540 nm.

##### 2.5.1.2 Cytotoxic effect of hydrogel by direct contact assay

To assess the cytocompatibility of nanosponge-integrated hydrogel by Direct Contact Assay, hydrogel discs (1 cm diameter) were placed in direct contact with fibroblast monolayers for 24 h. Following incubation, MTT reagent was added, followed by DMSO lysis. Absorbance was measured at 540 nm.

#### 2.5.2 *In Vitro* wound healing activity by scratch wound healing assay

Fibroblasts were cultured in 6-well plates to 90% confluence. Linear scratches were introduced using a sterile pipette tip. The cells were then treated with Taila (12.5 μg/mL) or hydrogel discs (5 mm diameter). Phase-contrast images of wound closure were captured at 0, 24, 48, and 72 h using ×4 magnification. Wound area was quantified using ImageJ software (Labovitz et al., 2026; Vašinková et al., 2025; Torabi et al., 2023).

#### 2.5.3 Determination of collagen deposition assay by sirius red staining method

Fibroblasts were cultured for 10 days in the presence of *taila* or the hydrogel. The amount of collagen synthesised was measured using sirius red staining. After centrifuging and eluting the bound dye with NaOH, the absorbance of the supernatant was measured at 530 nm.

#### 2.5.4 Antimicrobial study by agar well diffusion method

Petriplates containing 20 mL Muller Hinton Agar Medium were seeded with bacterial culture of *Pseudomonas aeruginosa*, *Streptococcus mutans, Esherichia coli and Staphylococcus aureus* (Al-Hamad et al., 2025) (growth of culture adjusted according to McFarland Standard, 0.5%). Wells of approximately 10 mm was bored using a well cutter and different concentrations of sample such as 250 μg/mL, 500 μg/mL and 1000 μg/mL were added. The plates were then incubated at 37 °C for 24 h. The antibacterial activity was assayed by measuring the diameter of the inhibition zone formed around the well (NCCLS, 1993). Streptomycin was used as a positive control.

## 3 Results

### 3.1 Characterization of *Vranaviropana taila*

The prepared *taila* is an oily, pale-yellow, semi-solid substance with a characteristic aroma and bitter taste. The physicochemical parameters of the *taila* are shown in Table 2. TLC and HPTLC fingerprinting confirmed the successful extraction of bioactive phytoconstituents (Joo et al., 2025) from *Snuhi* and *Arka* latex into the lipid base, revealing the presence of compounds including terpenoids, flavonoids, and steroids.

**TABLE 2:** Physicochemical parameters of *Vranaviropana taila*.

Parameter	Value
Loss on drying (%)	0.06
Specific gravity	0.92
Acid value (mgKOH/g)	8.67
Iodine value (g/100 g)	106.15
Refractive index (25 °C)	1.4678
Peroxide value (meq/kg)	1.37
HPTLC analysis	
Track 1 (5.0 µL, 15.0 mm) – No. of peaks	10
Track 2 (5.0 µL, 30.0 mm) – No. of peaks	11
Track 3 (5.0 µL, 45.0 mm) – No. of peaks	10

#### 3.1.1 TLC (Thin layer chromatography)

#### 3.1.2 HPTLC (High Performance Thin Layer Chromatography)

In TLC and HPTLC analysis of *Vranaviropana taila*, though no major spots appear at 254 nm, evaluation at 366 nm shows five distinct spots, which includes a prominent fluorescent orange band at Rf 0.56. This indicates the presence of flavonoid compounds. After derivatization, visible light discloses six spots in which the characteristic purple (Rf 0.54, 0.72, 0.76) and grey (Rf 0.62) bands strongly show the presence of phenolic compounds and potentially terpenes. Furthermore, a distinct yellow band at Rf 0.87 reveals the presence of flavonoids. Finally, HPTLC successfully determines 10 to 11 distinct peaks, establishing a consistent phytochemical profile of fluorescent bioactive constituents (Table 2).

### 3.2 Characterization of *taila*-loaded nanosponges

#### 3.2.1 Scanning electron microscopy

**FIGURE 4:**
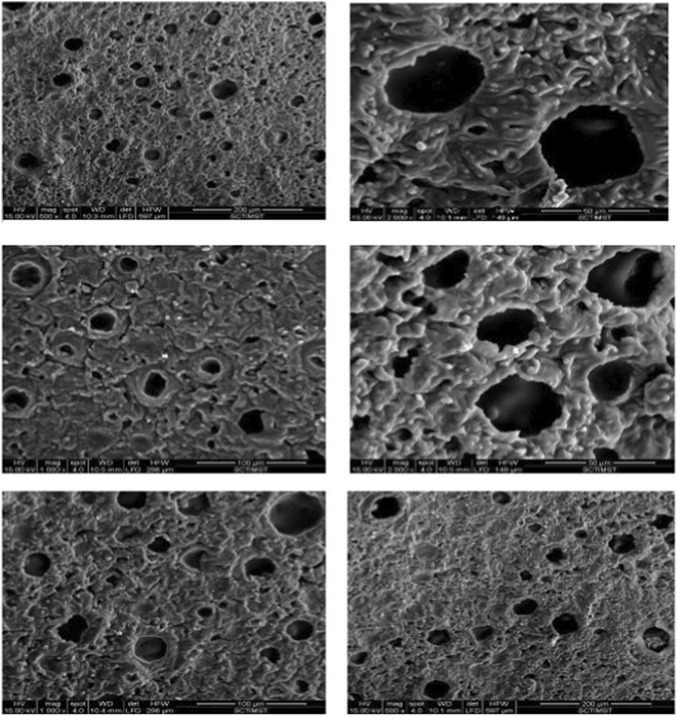
Scanning electron microscopy (SEM) images of the prepared nanosponges (gold-sputter coated and examined at appropriate accelerating voltage and magnifications) revealing a spherical morphology with a highly porous surface architecture indicative of successful nanosponge formation.

SEM analysis revealed that the taila-loaded nanosponges were predominantly spherical and exhibited a porous surface architecture. This morphology is indicative of a high surface area suitable for encapsulation and sustained release of phytoconstituents (Figure 4).

#### 3.2.2 Dynamic light scattering

DLS analysis demonstrated that the taila-loaded nanosponges possessed a uniform size distribution, with an average particle size of 461.3 nm and a narrow polydispersity index (PDI) of 0.061. The measured Zeta potential was −6.96 mV. Eventhough this value is low for purely electrostatic stabilization, the formulation keeps strong colloidal stability through a dual mechanism, which relies on the steric hindrance provided by the bulky polymeric matrix of the nanosponge in combination with this mild electrostatic repulsion.

#### 3.2.3 X-ray diffraction of nanosponges

**FIGURE 5:**
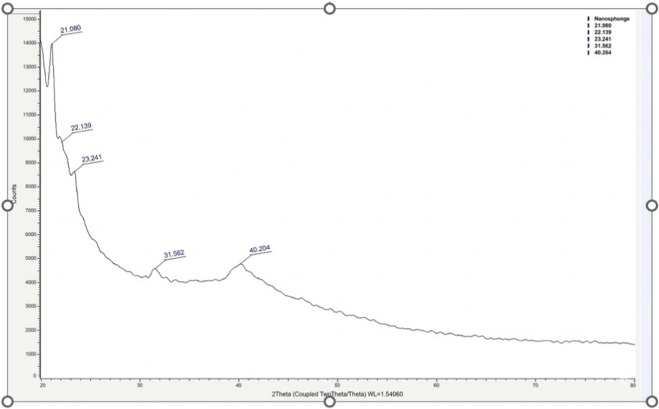
X-ray diffraction (XRD) pattern of the prepared nanosponges recorded using Cu Kα radiation (λ = 1.5406 Å) over a 2θ range of 20°–80°, demonstrating characteristic diffraction peaks indicative of their structural and crystallinity profile.

XRD analysis (Semenova et al., 2022) revealed a transition from a crystalline to an amorphous pattern in the taila-loaded nanosponges when compared to plain ethyl cellulose. This change indicates the successful encapsulation of the oil within the polymer matrix and a loss of the original crystalline structure (Figure 5).

#### 3.2.4 Encapsulation efficiency (EE%) and drug loading (DL%)

The synthesized nanosponges exhibited a high entrapment capacity with an estimated encapsulation efficiency (EE%) of 82.0% and a drug loading (DL%) of 75.5%, calculated based on a total recovered yield of 2.00 g and an initial input of 1.84 g of *Vranaviropana taila*.

### 3.3 Characterization of nanosponge-loaded hydrogel

#### 3.3.1 Swelling behavior

In phosphate-buffered saline (pH 7.4), the nanosponge-loaded hydrogel exhibited a swelling index of 57.5%. The hydrogel sample showed a dry weight of 0.2 g and a swollen weight of 0.315 g and an equilibrium swelling value of 0.115 g. While maintaining its structural integrity, the hydrogel demonstrated sufficient hydration and water absorption capacity, indicating its suitability for providing a moist wound-healing environment.

#### 3.3.2 Spreadability

The formulation exhibited a spreadability value of 6.8 g cm/s, indicating ease of topical application, appropriate flow characteristics and uniform consistency for wound-healing use.

#### 3.3.3 Gelling capacity

The formulation formed a stable gel within 35 s. It maintained structural integrity for more than 6 h. This confirms effective gelation and successful incorporation of nanosponges within the hydrogel matrix.

#### 3.3.4 *In Vitro* drug release

By mimicking skin and wound exudate conditions, diffusion studies conducted in PBS at both pH 6.8 and 7.4, demonstrated a gradual and controlled release of phytoconstituents (Arranz-Romera et al., 2021) over a 6-h period (Table 3). These results confirm the capacity of hydrogel for sustained delivery of bioactive components under physiological conditions. The hydrogel confirms its capacity for sustained delivery, evidenced by a controlled profile that prevents rapid dose dumping. This sustained behavior is mathematically validated by the Higuchi kinetic model (R2 > 0.96), indicating that the release mechanism is primarily governed by diffusion through the hydrogel matrix under physiological conditions. Drug release kinetics were evaluated at 37 °C ± 0.5 °C using phosphate buffers (pH 6.8 and 7.4) to simulate physiological conditions, while sink conditions were maintained by replacing 1 mL aliquots from a 9 mL medium to ensure rigorous spectrophotometric quantification of the sequestered oil fraction.

**TABLE 3:** *In vitro* drug release profile of *taila*-loaded hydrogel.

7 Time (h)	Absorbance @ pH 6.8	Absorbance @ pH 7.4
0	0.0267	0.0320
1	0.0542	0.0797
2	0.0613	0.0843
3	0.0779	0.0928
4	0.0804	0.0998
5	0.0896	0.1080
6	0.1042	0.1121

**FIGURE 6:**
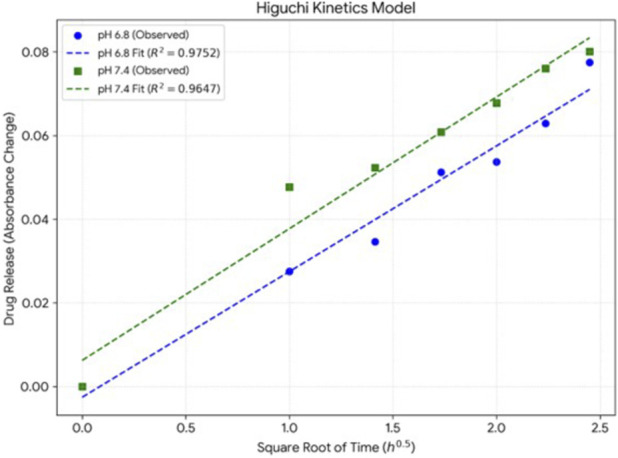
*In vitro* drug release profile of the nanosponge-loaded hydrogel evaluated using a dialysis membrane diffusion method in phosphate buffer (pH 6.8 and 7.4) at 37 °C ± 0.5 °C under continuous stirring, with release kinetics analyzed according to the Higuchi model.

As evidenced by the gradual and controlled release profile at both pH 6.8 and 7.4, the *in-vitro* drug release study confirmed the capacity of the hydrogel to provide sustained delivery of bioactive components under physiological conditions. Conventional *taila* is known to exhibit poor diffusion and rapid initial release. On the other hand, the hydrated polymeric matrix modulates diffusion of lipophilic constituents and enhances their availability at the wound site. The improved release behaviour of the hydrogel can be attributed to the hydrated polymeric matrix. A linear relationship was observed between cumulative drug release and the square root of time which was demonstrated by the Higuchi kinetic model, that indicates a diffusion-controlled release mechanism, which supports the superior sustained-release performance of the hydrogel formulation (Figure 6).

### 3.4 *In Vitro* study results

#### 3.4.1 Cytotoxicity assessment

Using L929 fibroblasts, the cytocompatibility of *Vranaviropana taila* and the nanosponge-integrated hydrogel was evaluated, and both treatments were found to be highly cytocompatible. A 91.96% cell viability was reported when L929 fibroblasts cells were exposed to the nanosponge-loaded hydrogel via direct contact assay. No detectable cytotoxic effects or adverse morphological alterations were observed, indicating excellent cytocompatibility. A high cell viability of >88% was reported, when L929 fibroblasts were treated with *Vranaviropana taila* (at 12.5 μg/mL, the concentration selected based on IC_50_ determination) in MTT assay. Both formulations demonstrated high viability relative to untreated controls (baseline 100%).

#### 3.4.2 *In vitro* wound healing efficacy

Compared to both the *taila* and the control (cells treated without sample) via two assays: fibroblast migration and collagen deposition, the nanosponge-integrated hydrogel demonstrated significantly enhanced wound healing activity.

**FIGURE 7:**
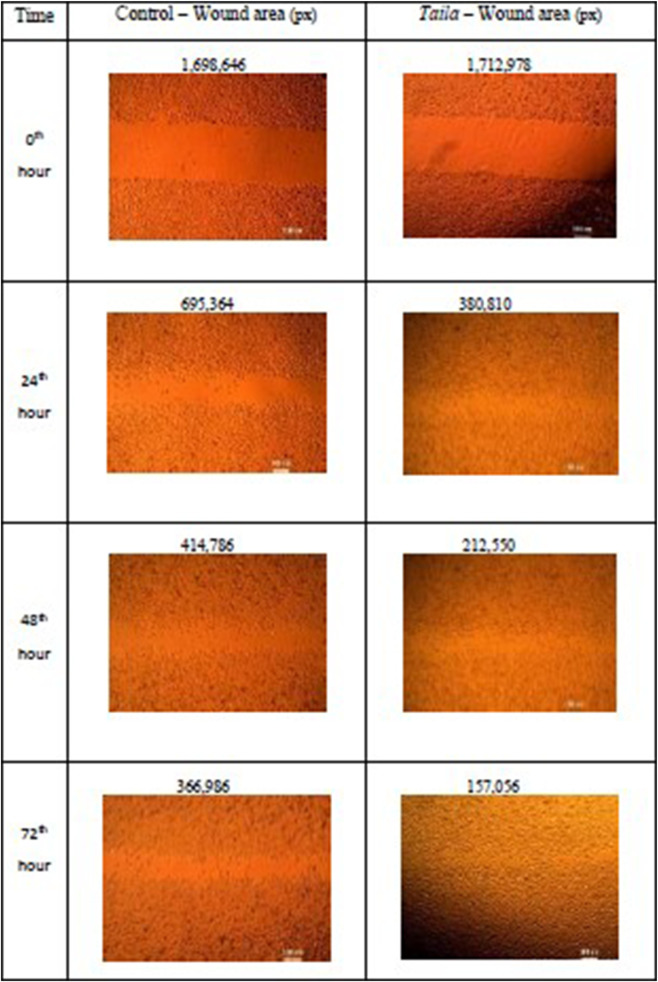
Representative photomicrographs of scratch wound assay performed in L929 fibroblast cells treated with Vranaviro pana taila, showing time-dependent wound closure (0–72 h) compared to control, with wound area quantified using image analysis software and expressed in pixels.

**FIGURE 8:**
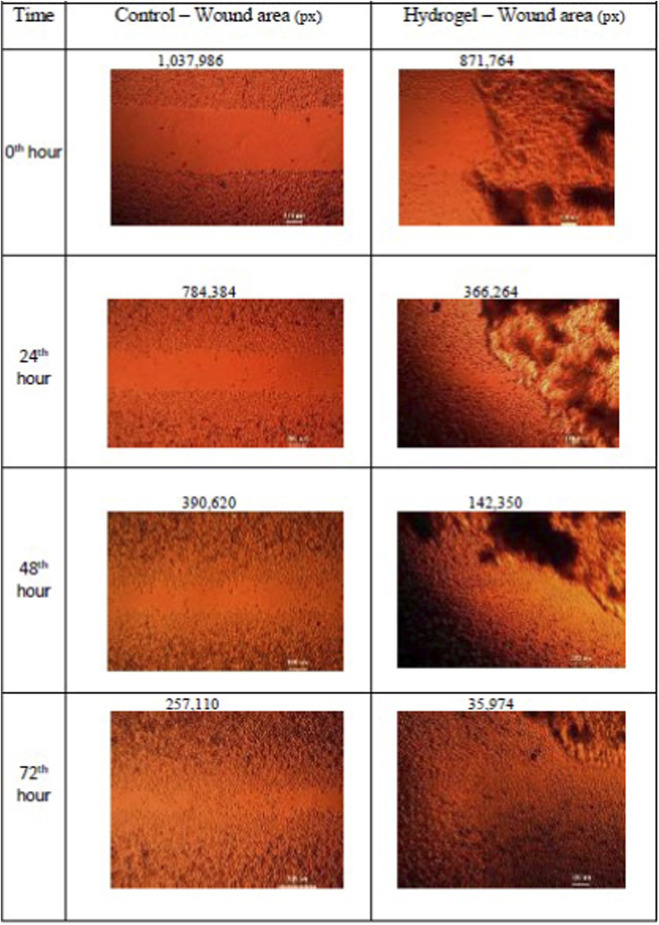
Representative photomicrographs of scratch wound assay in L929 fibroblast cells treated with nanosponge-loaded hydrogel, demonstrating time-dependent wound closure (0–72 h) compared to control, with wound area quantified by image analysis and expressed in pixels.

**FIGURE 9:**
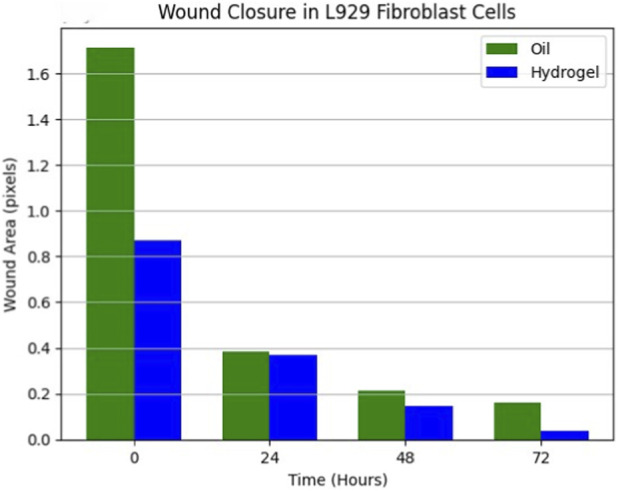
Quantitative analysis of scratch wound healing assay in L929 fibroblast cells treated with Vranaviro pana taila and nanosponge-loaded hydrogel, showing percentage wound closure calculated from initial wound area measurements over 72 h using image analysis software, with enhanced closure observed for the hydrogel formulation (∼96.5%) compared to taila (∼89.4%) at 72 h.

In Scratch wound healing Assay, after 72 h, the nanosponge-loaded hydrogel group exhibited the most effective wound closure at 96.5%. This was markedly superior to the *taila* group, which achieved 89.4% closure. These findings indicate that the hydrogel formulation significantly accelerates fibroblast migration compared to the oil (Figures 7–9; Table 4).

**TABLE 4:** Wound closure in L929 fibroblast cell treated with oil and hydrogel.

Oil			Hydrogel		
Time interval	Samples	Wound area (px)	Time interval	Samples	Wound area (px)
0th hour	Control	1,698,646	0th hour	Control	1,037,986
​	Oil	1,712,978	​	Hydrogel	871,764
24th hour	Control	695,364	24th hour	Control	784,384
​	Oil	380,810	​	Hydrogel	366,264
48th hour	Control	414,786	48th hour	Control	390,620
​	Oil	212,550	​	Hydrogel	142,350
72nd hour	Control	366,986	72nd hour	Control	257,110
​	Oil	157,056	​	Hydrogel	35,974

#### 3.4.3 Collagen deposition

**FIGURE 10:**
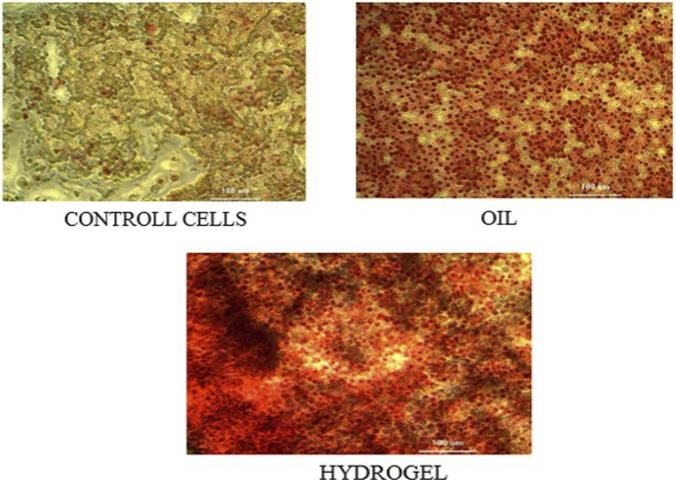
Representative photomicrographs showing collagen deposition in L929 fibroblast cells under control, Vranaviro pana taila (oil), and nanosponge-loaded hydrogel treatment conditions, visualized after collagen-specific staining and observed under light microscopy to assess extracellular matrix formation.

**FIGURE 11:**
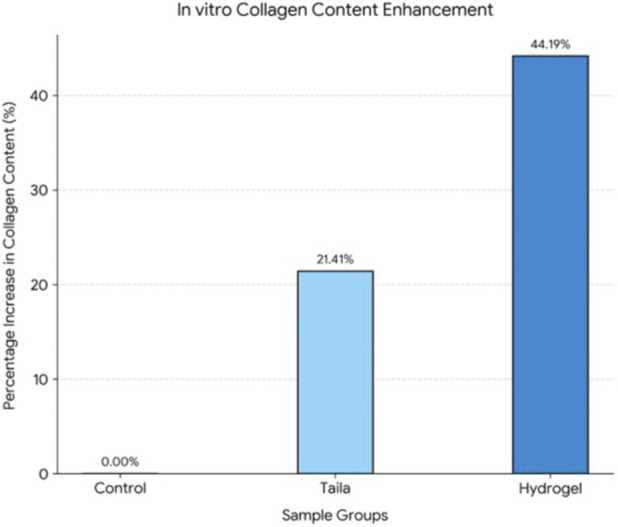
Quantitative estimation of collagen deposition in L929 fibroblast cells following treatment with Vranaviro pana taila (oil) and nanosponge-loaded hydrogel, expressed as percentage increase relative to untreated control and determined.

The pro-regenerative effect which was quantified by measuring collagen synthesis, showed a clear advantage for the hydrogel formulation. Nanosponge-integrated Hydrogel induced a 44.19% increase in collagen production. *Vranaviropana taila* induced a 21.41% increase in collagen production and the control served as the baseline with 0% increase. The enhanced collagen deposition observed with the hydrogel indicates a superior promotion of extracellular matrix remodeling, supporting its potential for accelerated wound healing (Figures 10, 11; Table 5).

**TABLE 5:** Showing Percentage increase in collagen content.

Samples	OD 1	OD 2	OD 3	Average OD	Percentage increase in collagen content
CONTROL	0.1705	0.1788	0.1765	0.1753	0
TAILA	0.2252	0.2298	0.2142	0.2231	21.41363
HYDROGEL	0.3122	0.3172	0.3129	0.3141	44.18975

#### 3.4.4 Statistics

The quantitative evaluation of collagen deposition was conducted with three independent experimental replicates (n = 3) per group. The optical density (OD) outcomes are reported as mean ± standard deviation (SD). The recorded mean OD values were 0.175 ± 0.004 for the untreated control, 0.223 ± 0.008 for the *Vranaviropana taila* group, and 0.314 ± 0.003 for the nanosponge-integrated hydrogel formulation. Statistical significance was evaluated using a one-way analysis of variance (ANOVA) followed by Tukey’s post-hoc test. The statistical analysis confirmed that the nanosponge-integrated hydrogel induced a highly significant enhancement in collagen deposition compared to both the untreated baseline control (p < 0.001) and the plain *taila* treatment (p < 0.001).

#### 3.4.5 Antimicrobial activity

**FIGURE 12:**
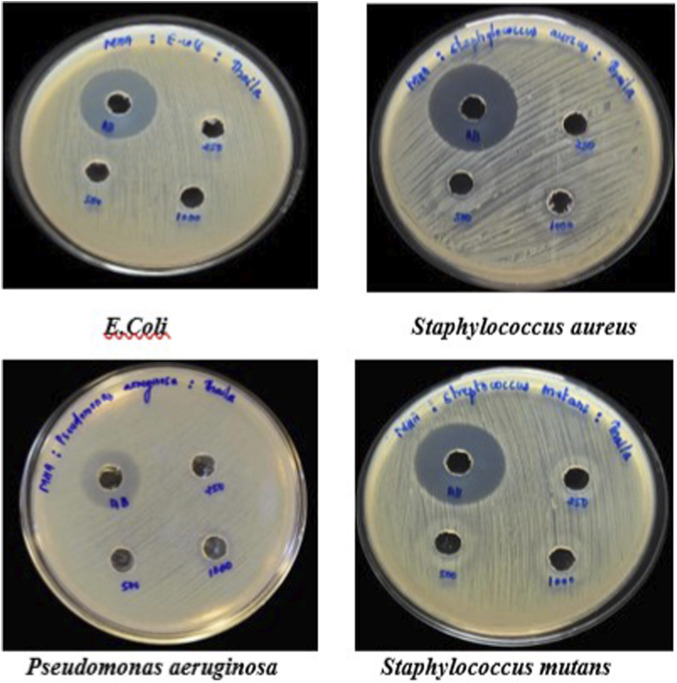
Antimicrobial activity of Vranaviro pana taila evaluated by agar well diffusion assay against *Escherichia coli*, *Staphylococcus aureus*, *Pseudomonas aeruginosa*, and *Streptococcus mutans*, with zones of inhibition measured in millimeters after incubation at 37 °C for 24 h.

**FIGURE 13:**
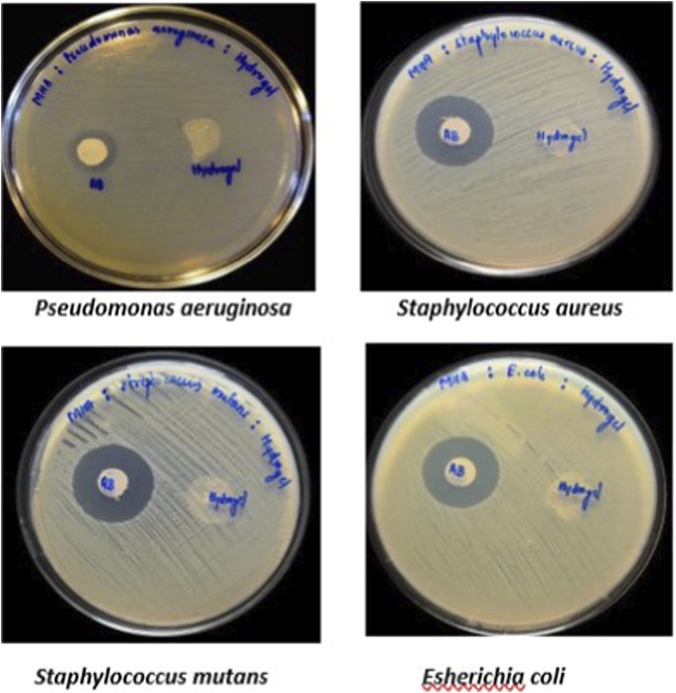
showing antimicrobial activity of Hydrogel - can u add sufficient experimental detail for frontiers in pharmacology in one brief sentence.

**FIGURE 14:**
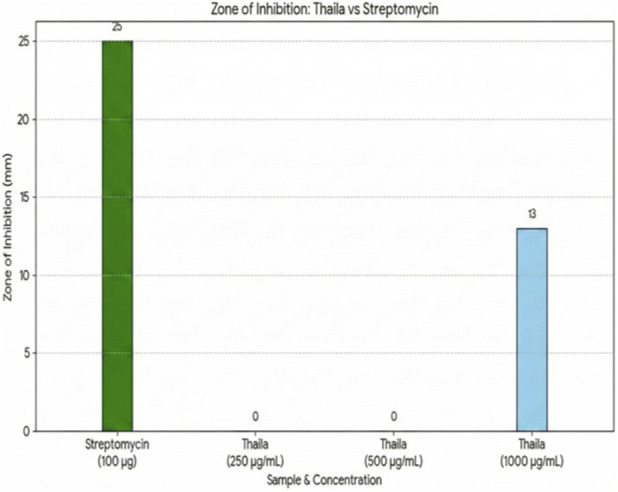
Antimicrobial activity of Vranaviro pana taila against *Pseudomonas aeruginosa* determined by agar well diffusion assay at different concentrations (250–1,000 μg/mL), with streptomycin (100 µg) as positive control, and zones of inhibition measured in millimeters after 24 h incubation at 37 °C.

The taila produced an inhibition zone of 13 mm against *Pseudomonas aeruginosa*, while that of the standard drug was 25 mm. No measurable inhibition zones were detected against the other tested organisms or with the hydrogel formulation (Figures 12–14; Table 6).

**TABLE 6:** Showing antimicrobial effect of *taila* against *Pseudomonas aeruginosa*.

Sample	Concentration (μg/mL)	Zone of inhibition (mm)
Streptomycin	100	25
*Taila*	250	0
	500	0
	1,000	13

## 4 Discussion

Ayurvedic medicated oils (*taila*) have a long history of use in wound management, appraised for their anti-inflammatory and regenerative properties. However significant formulation challenges like greasy nature, low water solubility and poor dermal adherence that leads to difficult application and compromised patient compliance hinder their clinical translation into primary care. This study sought to overcome these limitations by modifying *Vranaviropana taila* into an ethyl cellulose-based nanosponge-integrated Carbopol hydrogel.

This nano-integration provides critical mechanistic advantages. The primary challenge is the delivery of the lipophilic *taila*. By encapsulating the oil within porous, spherical nanosponges, as confirmed by SEM imaging, the physicochemical properties of *Vranaviropana taila* are fundamentally improved. The XRD analysis, which showed a loss of crystallinity, demonstrated successful encapsulation and amorphization of the oil within the polymer matrix. This nano-encapsulation improves the solubility and stability of the oil. High encapsulation efficiency (82.0%) and substantial drug loading capacity (75.5%) confirm the nanosponge matrix as a highly efficient carrier for delivering therapeutically relevant doses of Vranaviropana taila.

The larger nanoscale particle size of 461.3 nm, is expected and is attributed to the encapsulation of *Vranaviropana taila*. The incorporation of a highly viscous, multicomponent, complex oil naturally expands the internal porous matrix of the ethyl cellulose nanosponges, resulting in a larger hydrodynamic diameter compared to unloaded carriers. The SEM images provide clear morphological evidence, confirming the spherical, porous nature of the nanosponges and visually confirming the particle size range reported by the DLS analysis.

For systems relying purely on electrostatic repulsion, a zeta potential of −6.96 mV is relatively low. But the colloidal stability of our formulation is primarily governed by steric stabilization. In our formulation, the nanosponges are formulated using ethyl cellulose, a non-ionic, highly branched polymer, where stability is achieved through steric hindrance rather than electrostatic charge. This bulky polymeric network creates a physical barrier that prevents particles from approaching each other closely enough to induce van der Waals force-driven aggregation. This explains why the formulation remains physically stable inspite of a near-neutral surface charge.

Furthermore, this platform enhances penetration, retention, and local bioavailability. The nanosponge system, combined with the bioadhesive nature of the carbopol hydrogel, localizes the active phytoconstituents at the wound site. Unlike *Vranaviropana taila*, which can be easily displaced, the hydrogel holds the nanocarriers in place, allowing them to provide a sustained release of their payload over 6 hours, as shown by our *in vitro* release data. This sustained, localized delivery ensures a consistent therapeutic concentration of the active components of the taila directly at the site of action.

In promoting epithelialization and extracellular matrix (ECM) formation the hydrogel matrix plays a crucial therapeutic role. The desirable swelling behavior of hydrogel allows it to absorb exudate while maintaining a moist wound environment, which is essential for facilitating cell migration.

A similar work was conducted by Aldawsari et al. (2015), in which, localized therapeutic efficacy, stability and solubility was significantly improved by encapsulating lipophilic bioactives within nanosponges and incorporating them into a carbopol hydrogel. Like their observations, the sustained *in-vitro* release profile, and the loss of crystallinity on XRD in our study confirm successful nano-encapsulation and diffusion-controlled release from the hydrogel matrix, which leads to prolonged bioavailability at the site of application. Furthermore, the combined effects of hydrogel-induced moisture maintenance and nanosponge-mediated retention observed in both studies support the role of this platform in extracellular matrix formation, enhancing epithelialization, and overall wound healing outcomes.

Our *in vitro* findings supported this mechanism. The nanosponge-loaded hydrogel group exhibited the most effective wound closure of 96.5% which is superior to the taila group, that achieved 89.4% closure. This suggests that the moist environment, combined with the sustained release of bioactives, accelerates the migration of fibroblast which are responsible for depositing new collagen, the scaffold for tissue regeneration. The results of the study showed that the hydrogel induced a 44.2% increase in collagen deposition, more than double the effect of oil (21.41%). The hydrogel optimizes the physical environment for cell migration and the nanosponges provide a continuous therapeutic stimulus that explains the superior regenerative outcomes.

In another study conducted by Mo et al. (2025), it was observed that nanohybrid hydrogels accelerate tissue remodeling and fibroblast migration, by providing a moist microenvironment and sustained bioactive release. These study results summarized that the enhanced wound closure and collagen deposition with the nanosponge-loaded hydrogel are consistent. Like the mechanisms highlighted in their review, the combination of nano-carriers and hydrated hydrogel matrix in the present study creates favorable biochemical and physical indications that promote collagen synthesis and fibroblast activity. Collectively, these corresponds support the superior regenerative outcomes of the nanosponge-integrated hydrogel observed in our study and reinforce its translational relevance for advanced wound-healing applications.

Cytotoxicity studies showed that both the *taila* (>88% viability) and the final hydrogel formulation (>91% viability) were highly cytocompatible. This supports the traditional safety claims of *Snuhi* and *Arka* latex preparations. Overall, these findings demonstrate that integrating a classical Ayurvedic formulation with modern nanobiomaterials can successfully enhance therapeutic performance by overcoming key limitations in usability and drug delivery. This approach retains the medicinal value of the *taila* while improving its clinical potential, offering a promising, cost-effective wound care solution suitable for primary healthcare settings.

The formulation exhibited activity against *P. aeruginosa*, a major, therapeutically challenging pathogen in chronic wounds. It may be due to its inherent and acquired antibiotic resistance, suggesting its potential as an alternative or adjunct therapeutic option. The absence of activity against other organisms like *Staphylococcus aureus*, *Staphylococcus mutans* and *Esherichia coli* and lack of inhibition by the hydrogel may be attributed to limited diffusion related to beeswax content and the predominance of fat-soluble active constituents, while the structural barrier of hydrogel itself may contribute to the prevention of infection by restricting microbial penetration.

Shahrour et al. (2025) illustrated that, despite its high intrinsic and acquired resistance, hydrogel-based systems can effectively target *P. aeruginosa* in chronic wound environments. Rather than broad-spectrum diffusion, it may be primarily through localized retention. In the present study the limited activity against other organisms reflects formulation-dependent diffusion constraints and the role of the hydrogel matrix as a physical barrier that prioritizes infection containment over nonspecific antimicrobial action. This aligns with the findings of the study conducted by Shahrour et al. (2025).

## 5 Limitations

Nanosponge-integrated *Vranaviropana taila* hydrogel has demonstrated *invitro* wound healing activity, by providing important preliminary insights into the wound-healing potential of the formulation like cytocompatibility and collagen deposition. Despite these promising results, the present study, cannot fully replicate the complex physiological processes involved in wound repair, such as angiogenesis, immune responses and inflammatory response.

Therefore, the present findings are limited to a simplified biological system. Further preclinical *in vivo* animal studies are required to evaluate crucial parameters such as anti-inflammatory mechanisms, biodegradation, systemic safety, pharmacokinetics, tissue regeneration dynamics, and infection control efficacy under physiological stress.

Furthermore, no clinical trials have yet been conducted to validate the therapeutic efficacy, safety, and pharmacokinetic action of the formulation in humans. Clinical validation is essential to establish therapeutic consistency, safety and efficacy, before the formulation can be recommended for wound management in primary healthcare settings.

Additionally, the present study is limited by insufficient chemical standardization of the complex herbal formulation; the phytopharmacological analysis provides only qualitative identification of flavonoids and phenols. Future studies must include quantitative determination of total phenolic and flavonoid content, chromatographic fingerprinting (such as HPLC/LC-MS), and the identification of specific bioactive markers to ensure scientific rigor and batch-to-batch reproducibility. Finally, essential pharmaceutical characterizations, including detailed viscosity profiling and long-term stability assessments of the hydrogel, remain to be established.

## 6 Further scope of the study

Before progressing to advanced biological testing, future research must prioritize the chemical standardization which includes the advanced chromatographic fingerprinting such as HPLC/LC-MS. Furthermore, comprehensive pharmaceutical characterizations, including detailed viscosity profiling and long-term stability assessments of the hydrogel, must be established to guarantee a robust product.

Further research in this area should focus on translating the encouraging *in vitro* findings of the nanosponge-integrated *Vranaviropana taila* hydrogel into *in vivo* and clinical investigations which comprehensively validate its wound-healing efficacy and safety. These studies are necessary to assess key efficacy endpoints such as tissue regeneration, angiogenesis, re-epithelialization, and inflammatory modulation under physiological conditions. Simultaneously, dose optimization studies are required to determine the most effective therapeutic concentration. These studies will further help to determine potential systemic effects of the formulation, pharmacokinetics and *in vivo* biocompatibility.

Subsequently, controlled clinical trials involving human subjects with acute or chronic wounds are essential to establish therapeutic efficiency, biocompatibility and patient tolerance. These trials will be necessary to establish definitive therapeutic efficacy, patient tolerance, and safety, including the potential for allergic reactions. Such trials, integrating pharmacological validation and cost-effectiveness assessments, will be critical for supporting its large-scale adoption within primary healthcare systems, particularly in resource-limited regions. These animal and clinical studies form the vital bridge between laboratory success and true clinical applicability, ensuring the safe and evidence-based use of this Ayurvedic nanohydrogel in primary healthcare.

## 7 Conclusion

In this study the traditional Ayurvedic formulation *Vranaviropana taila* is modified into an ethyl cellulose nanosponge-based hydrogel by creating a bioactive wound dressing suitable for primary healthcare applications. Nanosponge-integrated *Vranaviropana taila* hydrogel exhibited enhanced wound healing activity through improved tissue regeneration and accelerated fibroblast migration along with superior physicochemical stability and biocompatibility compared to conventional preparations. The traditional knowledge in classics is validated by the presence of phenolic and flavonoid-rich phytoconstituents that supports its antioxidant and tissue-repair potential. Nanosponge-integrated *Vranaviropana taila* hydrogel demonstrated excellent wound-healing performance and formulation stability. This formulation prepared by cost-effective methods is an accessible solution for community-level wound management in primary healthcare. By integrating Ayurvedic phytopharmacology with nanotechnology, this work bridges traditional medicine and modern biomedical innovation, offering a sustainable path toward developing clinically relevant, affordable wound care systems for use in primary healthcare and resource-limited settings.

However, it must be highlighted that these findings are strictly preliminary and based on *in vitro* models. Accordingly, the current projections regarding the cost-effectiveness, scalability and translational readiness of the formulation remain uncertain at this stage and require rigorous validation through comprehensive clinical trials, extensive *in vivo* studies, formal economic analyses and long-term stability testing before definitive clinical applicability can be established.

## Data Availability

The original contributions presented in the study are included in the article/supplementary material, further inquiries can be directed to the corresponding author.
